# Sexual Dimorphism in Circadian Physiology Is Altered in LXR*α* Deficient Mice

**DOI:** 10.1371/journal.pone.0150665

**Published:** 2016-03-03

**Authors:** Céline Feillet, Sophie Guérin, Michel Lonchampt, Catherine Dacquet, Jan-Åke Gustafsson, Franck Delaunay, Michèle Teboul

**Affiliations:** 1 University Nice Sophia Antipolis, Institute of Biology Valrose, 06108, Nice, France; 2 CNRS UMR 7277, 06108, Nice, France; 3 INSERM UMR 1091, 06108, Nice, France; 4 Metabolic Diseases Research, Institut de Recherches Servier, 92284, Suresnes, France; 5 Center for Nuclear Receptors and Cell Signaling, Department of Biology and Biochemistry, University of Houston, Houston, Texas, 77204–5056, United States of America; University of Fribourg, SWITZERLAND

## Abstract

The mammalian circadian timing system coordinates key molecular, cellular and physiological processes along the 24-h cycle. Accumulating evidence suggests that many clock-controlled processes display a sexual dimorphism. In mammals this is well exemplified by the difference between the male and female circadian patterns of glucocorticoid hormone secretion and clock gene expression. Here we show that the non-circadian nuclear receptor and metabolic sensor Liver X Receptor alpha (LXR*α*) which is known to regulate glucocorticoid production in mice modulates the sex specific circadian pattern of plasma corticosterone. *Lxrα*^*-/-*^ males display a blunted corticosterone profile while females show higher amplitude as compared to wild type animals. Wild type males are significantly slower than females to resynchronize their locomotor activity rhythm after an 8 h phase advance but this difference is abrogated in *Lxrα*^*-/-*^ males which display a female-like phenotype. We also show that circadian expression patterns of liver *11β-hydroxysteroid dehydrogenase type 1* (*11β-HSD1*) and *Phosphoenolpyruvate carboxykinase* (*Pepck*) differ between sexes and are differentially altered in *Lxrα*^*-/-*^ animals. These changes are associated with a damped profile of plasma glucose oscillation in males but not in females. Sex specific alteration of the insulin and leptin circadian profiles were observed in *Lxα*^*-/-*^ females and could be explained by the change in corticosterone profile. Together this data indicates that LXR*α* is a determinant of sexually dimorphic circadian patterns of key physiological parameters. The discovery of this unanticipated role for LXR*α* in circadian physiology underscores the importance of addressing sex differences in chronobiology studies and future LXR*α* targeted therapies.

## Introduction

In mammals, many molecular, cellular, physiological and behavioural processes show circadian (~24 h) oscillations synchronised to the external light/dark cycle. These circadian rhythms are under the control of a self-sustained internal clock present in nearly every cell. At the organism level, these clocks are organized hierarchically with at the top a central pacemaker located in the suprachiasmatic nuclei (SCN) of the hypothalamus that receives photic cues and in turn coordinates local clocks in the periphery. Peripheral clocks are entrained by the SCN through internal systemic synchronizers such as glucocorticoid hormones and body temperature and most probably other signals that remain to be identified. Although peripheral clocks display self-sustained oscillations at the single-cell level, at the organ and systemic levels they require an intact SCN clock to remain in phase [1, 2]. At the molecular level, the core mechanism of all cellular clocks is governed by a genetic network that integrates multiple time delayed negative and positive feedbacks loops [3]. The primary loop involves the two bHLH-PAS transcription factors CLOCK and BMAL1 which upon dimerization trigger transcription of the *Period (Per1-3)* and *Cryptochrome (Cry1/2)* clock genes. PER and CRY proteins then translocate to the nucleus where they in turn repress the CLOCK-BMAL1 transactivation [4]. The core clock mechanism also involves the nuclear receptors ROR(α,β,γ) and REV-ERB(α,β) which are direct CLOCK:BMAL1 targets and compete to activate or repress the transcription of the *Bmal1* and *Clock* genes respectively. This secondary loop provides robustness to the circadian oscillator and is critical for normal circadian behavior and physiology [5, 6]. In addition to these transcriptional mechanisms, the circadian molecular network is also extensively regulated post-translationally [7–9] as well as through chromatin remodelling [10, 11]. Oscillation of this increasingly complex network directs the rhythmic expression of downstream clock-controlled genes through transcriptional, post-transcriptional, translational and post-translational mechanisms [12, 13]. A recent meta-analysis of available genome wide circadian gene expression data has estimated that approximately 43% of mouse genes oscillate somewhere in the body yet with a significant tissue-specificity, thus highlighting the extent of circadian regulation in mammals [14].

Nuclear hormone receptors form a large family of proteins which function as ligand-inducible transcription factors involved in virtually all key biological processes and expectedly in many diseases. In addition to the REV-ERB and ROR receptors, a substantial number of these receptors have been implicated either in the core circadian clock mechanism or as transcriptional links between the clock gene network and clock-controlled processes [15]. The glucocorticoid receptor (GR) is for instance directly involved in the resetting of peripheral clocks by glucocorticoids [16]. The essential clock gene *Bmal1* is regulated by peroxisome proliferator activated receptors (PPARs) *α* and γ in the liver and the cardiovascular system respectively [17, 18]. Other examples include the estrogen receptor β, constitutive androstane receptor, short heterodimer partner and the estrogen related receptor *α* [19–22]. Many of these receptors have been implicated in the circadian regulation of metabolism [23]. Liver X receptor (LXR) *α* is another important metabolic nuclear receptor regulating cholesterol, fatty acids and glucose homeostasis [24]. A critical function of LXR*α* is to activate bile acid formation through upregulation of cholesterol catabolism by the CYP7*α*1 enzyme encoding gene. LXR*α* was suggested to be responsible for the circadian expression of the *Cyp7α1* gene through rhythmic activation by its endogenous ligands that include 22(R)-hydroxycholesterol, 24(S)-hydroxycholesterol, 27-hydroxycholesterol, and cholestenoic acid [25]. Interestingly, male *Lxrα*^*-/-*^ mice display adrenomegaly and increased corticosterone (CORT) secretion [26]. Given the pivotal role of glucocorticoids (GCs) in the synchronization of peripheral clocks, this observation suggests that although LXR*α* is not clock-controlled, it may play an unanticipated role in circadian physiology [27]. Notably, the mammalian hypothalamo-pituitary-adrenal (HPA) axis exhibits a marked and well documented sexual dimorphism [28]. In particular, females show higher mean levels and amplitude of corticosterone as compared to males. Given the impact of the loss of LXR*α* in the adrenals, we hypothesized that LXR*α* could contribute to such sex difference. To address this issue we analyzed the circadian physiology of LXR*α* deficient animals and obtained evidence that known and newly identified sex differences in circadian regulation are indeed altered in these animals.

## Results

### The sexual dimorphism of the circadian corticosterone pattern is differentially changed in male and female *Lxrα*^*-/-*^mice

**Fig 1:**
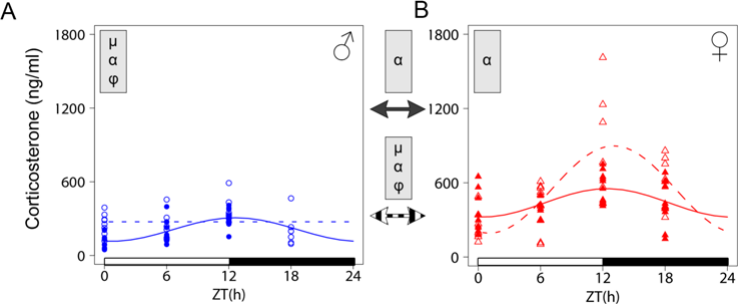
The sexually dimorphic circadian pattern of plasma CORT is differentially altered in male and female *Lxrα*^*-/-*^ mice. (A) Rhythm of plasma CORT was determined in wild type (plain line) and *Lxrα*^*-/-*^ (dashed line) male mice entrained to a LD12:12 cycle. (B) Same as in (A) except that mice were females. Cosinor based non-linear regression was used for curve fitting. Plotted data were from 10 mice for each experimental group. The ZT0 time point is double plotted for visualization purpose. White and black bars represent the light and dark phases, respectively. Statistically significant differences in cosine fitting parameters (p<0.05) between wild type and *Lxrα*^*-/-*^ mice or between male and female of the same genotype is indicated in the grey box at the top of the corresponding graph or between graphs (WT: plain arrow, *Lxrα*^*-/-*^: dashed arrow). µ, α and φ indicate a difference in mean level, amplitude and acrophase, respectively.

Plasma GCs levels show daily oscillations in mammals including mice with higher concentrations being observed at the end of the resting phase. Further, female mice display significantly higher mean CORT levels than males irrespective of the genetic background [29]. Of note a circadian pattern of CORT in females is observed in non estrus-synchronized females [29–31]. Based on the known implication of LXR*α* in adrenal corticosterone production, we measured plasma CORT levels at four time points around the clock in wild type (WT) and *Lxrα*^*-/-*^ male and non estrus-synchronized female mice entrained to an LD12:12 cycle. Expectedly, plasma CORT showed a robust daily variation in WT mice with an acrophase at the beginning of the night (Fig 1 and Table 1). Mean levels were significantly higher in females than in male WT mice (Fig 1 and Table 1). Loss of LXR*α* resulted in a significant and opposite effect in males as compared to females. Indeed, male *Lxrα*^*-/-*^ mice showed significantly higher mean CORT levels with no circadian variation as compared to WT mice (Fig 1 and Table 1). In sharp contrast, *Lxrα*^*-/-*^ females had similar mean CORT levels as compared to WT females but they displayed a 3 times higher amplitude. We also confirmed that the expression of glucocorticoid receptor (*GR*) did not show circadian rhythmicity [27]. We found that mean level of *GR* mRNA was lower in *Lxrα*^*-/-*^ males as compared to WT animals, consistent with the known downregulation of GR expression by its ligand [32]. We also found a small but significant difference between males and females (S1 Fig and S1 Table). We conclude from these observations that both the level and the circadian pattern of plasma CORT are differentially impacted by the LXR*α* mutation in males and females.

**Table 1:** Cosinor analysis of plasma CORT circadian pattern in WT and *Lxrα* ^*-/-*^ mice.

Group	Mean level (ng/ml)	Amplitude	Acrophase (ZT:min)
♂ WT	206.7 (181.4 ; 231.2)	94.9 (62.5 ; 129.0)	12:16 (10:49 ; 13:47)
♂ *Lxrα* ^*-/-*^	274.0 (235.8 ; 317.0)†	NSR	NSR
♀ WT	437.6 (388.7 ; 486.5)*	114.7 (62.5 ; 179.4)	12:24 (09:49 ; 14:52)
♀ *Lxrα* ^*-/-*^	545.4 (458.3 ; 634.3)*	356.6 (225.1 ; 492.1)*†	13:03 (11:52 ; 14:23)*

For each parameter measured, values are represented as median ± 95% bootstrap confidence intervals.

* indicates significant differences between females *vs* males

† indicates significant differences between *Lxrα^-/-^ vs* WT mice.

Circadian rhythmicity was considered significant for a *p*-value < 0.05; NSR, not significantly rhythmic.

### Time to reset after jetlag is decreased in *Lxrα*^*-/-*^males

**Fig 2:**
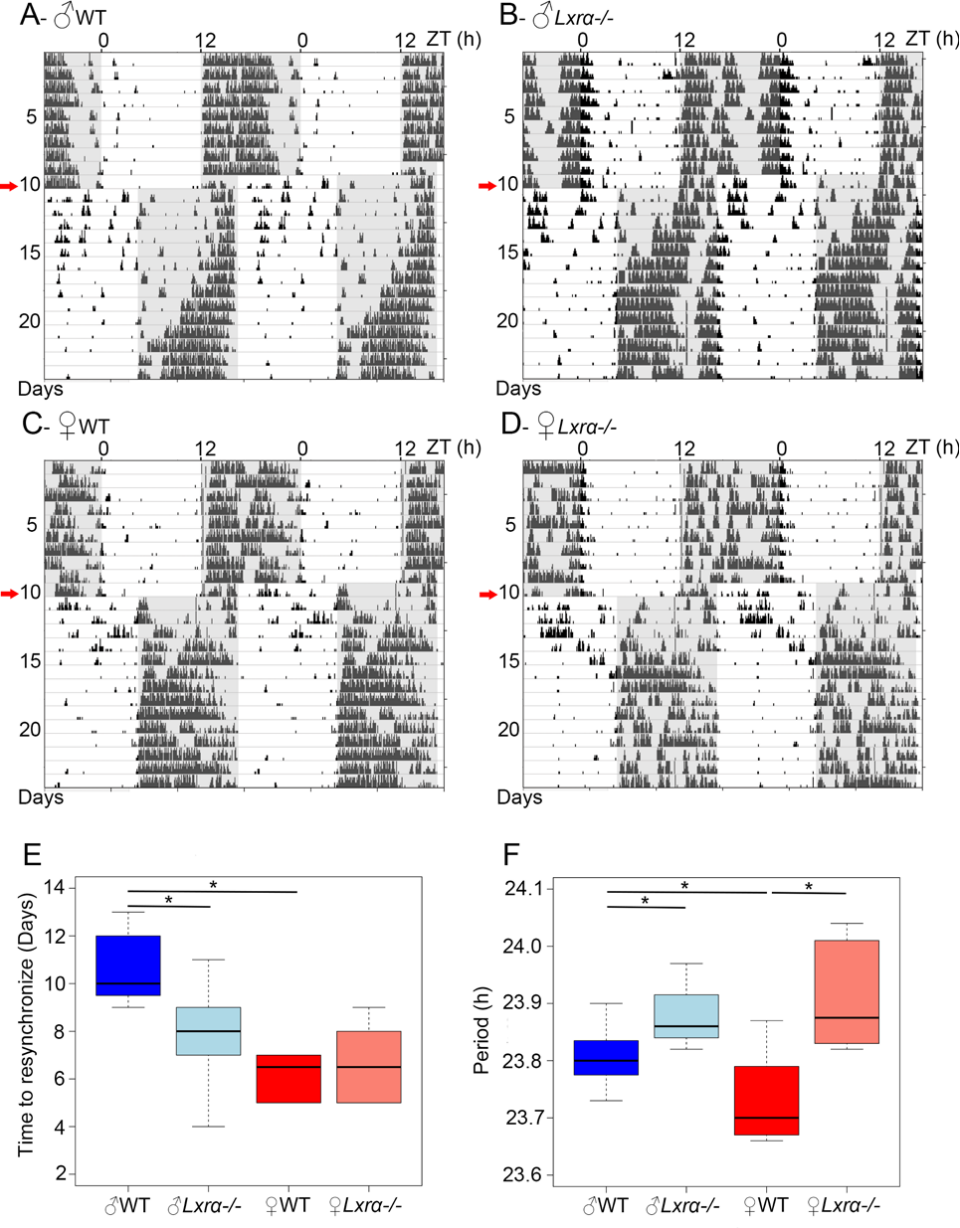
Resetting after jetlag differs between male and female in wild type but not in *Lxrα*^*-/-*^ mice. Wheel running activity was measured in animals entrained to a LD12:12 and then submitted to an 8-hours jetlag (red arrow). Representative actograms for male (A) and female (B) wild type mice and male (C) and female (D) *Lxrα*^*-/-*^ mice are shown. In A-D, the white and grey bars represent the light and dark phases respectively. Box and whiskers plots for resetting-time (E) and free-running period of wheel running activity (F) in male (n = 8) and female (n = 6) wild type mice and in male (n = 11) and female (n = 6) *Lxrα*^*-/-*^ mice. * indicates statistically significant differences (p<0.05) between groups.

Glucocorticoid hormones are well known to play a role in the resetting of the central clock after a phase shift in mammals [33, 34]. As the CORT pattern was differentially changed in male and female *Lxrα*^*-/-*^ mice, we investigated circadian pattern and resetting of the locomotor activity rhythm in these animals. Male and female *Lxrα*^*-/-*^ mice entrained to an LD 12:12 cycle and then released in constant darkness showed a similar level of total wheel running activity and a small, although significant, increase of their free running period length of locomotor activity as compared to WT animals (Fig 2F and Table 2). After re-entrainment to an LD 12:12 cycle, the animals were subjected to an 8-hours phase advance of the LD cycle; we observed a significant sexual dimorphism in WT animals with females requiring approximately 6 days to re-entrain with the new phase as compared to 10 days for males (Fig 2A, 2C and 2E and Table 2). This difference was abolished in *Lxrα*^*-/-*^ males which became significantly faster than their WT controls, and consequently were similar to females (Fig 2B, 2D and 2E, Table 2). Note that some *Lxrα*^*-/-*^ females tended to re-entrain with a combination of advances (onset) and delays (offset) of their activity patterns, rather than simply advancing activity onset. These results suggest that LXR*α* does not play a major role in the control of the endogenous period by the central circadian pacemaker but in contrast dramatically impacts on its resetting properties in males.

**Table 2:** Total daily activity, free-running period of locomotor activity and resynchronization time of WT and *Lxrα*^*-/-*^ mice.

Group	Daily activity (LD 12/12)	Period (DD, hours : min)	Resetting (days)
♂ WT	7736 (7048 ; 8294)	23:48 (23:47 ; 23:50)	10.00 (9.75 ; 12.00)
♂ *Lxrα* ^*-/-*^	6638 (5849 ; 8474)	23:52 (23:50; 23:55)†	8.00 (7.00; 9.00)†
♀ WT	6732 (5555 ; 8612)	23:42 (23:41 ; 23:46)*	6.50 (5.25 ; 7.00)*
♀ *Lxrα* ^*-/-*^	8097 (7384 ; 8681)	23:53 (23:50 ; 23:59)†	6.50 (5.25 ; 7.75)

Values are represented as median ± 95% bootstrap confidence intervals.

* indicates significant differences between males vs females

† indicates significant differences between WT *vs* Lxr*α*^-/-^ mice.

### Loss of LXR*α* differentially alters circadian gene expression in males and females

**Fig 3:**
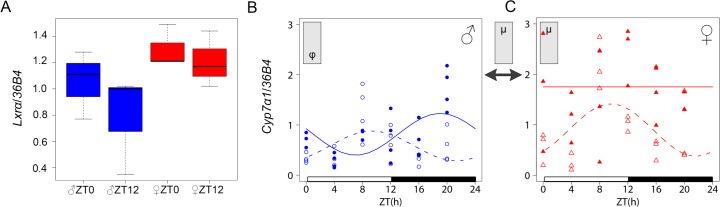
Analysis of *Lxrα* and *Cyp7α1* in liver. *Lxrα* mRNA expression determined at ZT0 and ZT 12 in WT males and females using qRT-PCR (A). Diurnal mRNA expression of liver *Cyp7α1* was compared in males (B) and females (C) using qRT-PCR in WT (plain line) and *Lxrα*^*-/-*^ mice (dashed line). For each time point, 3–4 mice were used. For the *Cyp7α1* analysis, cosinor-based non-linear regression was used for curve fitting. The ZT0 time point is double plotted for visualization purposes. Expression data were normalized to the constitutively expressed *36B4* mRNA. The white and black bars represent the light and dark phases, respectively. Statistically significant differences in cosine fitting parameters (p<0.05) between wild type and *Lxrα*^*-/-*^ mice or between male and female of the same genotype is indicated in the grey box at the top of the corresponding graph or between graphs (WT: plain arrow, *Lxrα*^*-/-*^: dashed arrow). µ, α and φ indicate a difference in mean level, amplitude and acrophase, respectively.

We first analysed the expression of *Lxrα* in liver and adrenals in WT mice and found no significant time of day- or sex- dependent variation confirming and extending previous data (Fig 3A, S2 Fig) [27]. Because *Cyp7α1* is a well established direct target of LXR*α*, that displays a mRNA circadian variation, we compared its expression in male and female *Lxrα*^*-/-*^ mice. Data shows that the *Cyp7α1* mRNA oscillates in WT males but not in females. Upon deletion of LXR*α* this pattern is phase advanced by approximately 10-h in males and became rhythmic in females with a similar phase (Fig 3B and 3C, Table 3).

**Table 3:** Cosinor analysis of *Cyp7α1* circadian expression in WT and *Lxrα*^*-/-*^ mice.

Gene	Group		Amplitude	Acrophase (ZT:min)
*Cyp7 α 1*	♂ WT	0.81 (0.64 ; 1.01)	0.41 (0.18 ; 0.70)	19:18 (16:22 ; 21:43)
	♂ *Lxrα*^*-/-*^	0.58 (0.45 ; 0.74*)*	0.30 (0.12; 0.52)	9.21 (6:24; 12:35)†
	♀ WT	1.83 (1.53 ; 2.15)*	NSR	NSR
	♀ *Lxrα*^*-/-*^	0.90 (0.64 ; 1.18)†	0.52 (0.20; 0.96)	9.37 (6:29; 12:33)

Values are represented as median ± 95% bootstrap confidence intervals.

* indicates significant differences between females *vs* males

† indicates significant differences between *Lxrα^-/-^ vs* WT mice.

Circadian rhythmicity was considered significant for a *p*-value < 0.05; NSR, not significantly rhythmic.

**Fig 4:**
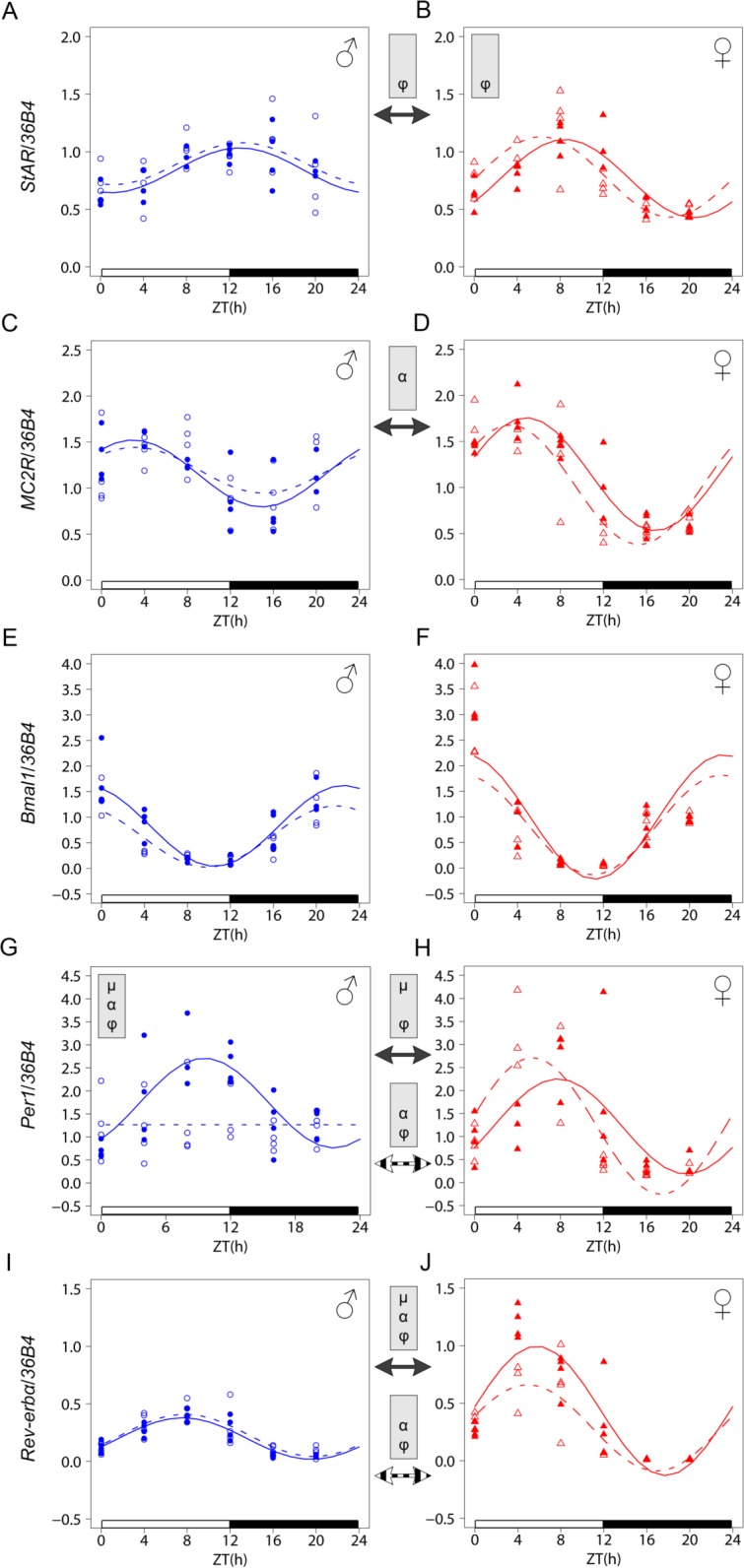
Analysis of circadian gene expression in the adrenals of WT and *Lxrα*^*-/-*^ mice. Diurnal mRNA expression of adrenal *StAR* (A, B), *MC2R* (C, D), *Bmal1* (E, F), *Per1* (G, H) and *Rev-erbα* (I, J) was compared using qRT-PCR in WT (plain line) and *Lxrα*^*-/-*^ mice (dashed line). For each time point, 3–4 mice were used. Cosinor-based non-linear regression was used for curve fitting. The ZT0 time point is double plotted for visualization purposes. Expression data were normalized to the constitutively expressed *36B4* mRNA. The white and black bars represent the light and dark phases, respectively. Statistically significant differences in cosine fitting parameters (p<0.05) between wild type and *Lxrα*^*-/-*^ mice or between male and female of the same genotype is indicated in the grey box at the top of the corresponding graph or between graphs (WT: plain arrow, *Lxrα*^*-/-*^: dashed arrow). µ, α and φ indicate a difference in mean level, amplitude and acrophase, respectively.

Based on the changes observed in LXR*α* deficient mice regarding the plasma CORT pattern, we investigated the expression profiles of the *Adreno-Cortico-Tropic-Hormone-receptor* (*MC2R*) and *Steroidogenic Acute Regulatory* (*StAR*) mRNA, two key determinants of CORT synthesis in the adrenals. They both display a circadian expression at the mRNA level in males and the rhythmic CORT pattern has been linked to the circadian expression of *StAR* [35]. We observed higher amplitude of the *MC2R* mRNA profile in females as compared to males irrespective of the genotype, suggesting that LXR*α* is unlikely to significantly modulate the response of the adrenal to ACTH (Fig 4A and 4B and Table 4). We found that the acrophase of *StAR* mRNA was advanced by 4 to 6 hours in females as compared to males, independently of the genotype (Fig 4C and 4D and Table 4). This sex difference was potentiated in the knockout animals as *Lxrα*^*-/-*^ females displayed an even earlier peak than their controls while *Lxrα*^*-/-*^ and WT males were not different.

**Table 4:** Cosinor analysis of circadian gene expression in the adrenals of WT and *Lxrα*^*-/-*^ mice.

Gene	Group	Mean level	Amplitude	Acrophase (ZT:min)
*StAR*	♂ WT	0.83 (0.78 ; 0.90)	0.20 (0.13 ; 0.28)	12:58 (11:17 ; 14:37)
	♂ *Lxrα* ^*-/-*^	0.90 (0.82 ; 0.98)	0.18 (0.08 ; 0.32)	13:09 (10:35 ; 15:58)
	♀ WT	0.77 (0.72 ; 0.82)	0.34 (0.27 ; 0.42)	8:26 (7:32 ; 9:16) *
	♀ *Lxrα* ^*-/-*^	0.78 (0.71 ; 0.85)	0.35 (0.26 ; 0.46)	6:14 (5:02 ; 7:26) †
*MC2R*	♂ WT	1.15 (1.06 ; 1.27)	0.36 (0.23 ; 0.50)	2:56 (1:14 ; 4:25)
	♂ *Lxrα*^*-/-*^	1.19 (1.07 ; 1.32)	0.25 (0.10 ; 0.45)	3:08 (0:08 ; 6:17)
	♀ WT	1.14 (1.07 ; 1.22)	0.61 (0.51 ; 0.73)*	4:49 (4:05 ; 5:32)
	♀ *Lxrα*^*-/-*^	1.03 (0.92 ; 1.15)	0.66 (0.50 ; 0.82)*	3:18 (2:23 ; 4:16)
*Per1*	♂ WT	1.74 (1.51 ; 1.97)	0.98 (0.64 ; 1.35)	9:34 (8:17 ; 10:53)
	♂ *Lxrα*^*-/-*^	1.27 (1.04 ; 1.49)†	NSR	NSR
	♀ WT	1.09 (0.86 ; 1.33)*	0.92 (0.61 ; 1.28)	6:53 (5:33 ; 8:10)*
	♀ *Lxrα*^*-/-*^	1.23 (0.95 ; 1.51)	1.50 (1.11 ; 1.90)	5:22 (4:23 ; 6:21)
*Bmal1*	♂ WT	0.83 (0.71 ; 0.97)	0.79 (0.61 ; 0.97)	22:32 (21:40 ; 23:25)
	♂ *Lxrα*^*-/-*^	0.62 (0.51 ; 0.74)	0.60 (0.46 ; 0.76)	21:47 (20:46 ; 22:48)
	♀ WT	1.00 (0.72 ; 1.26)	1.22 (0.84 ; 1.66)	23:11 (21:56 ; 00:21)
	♀ *Lxrα*^*-/-*^	0.84 (0.63 ; 1.08)	0.98 (0.67 ; 1.34)	23:00 (21:41 ; 00:17)
*Rev-erbα*	♂ WT	0.20 (0.17 ; 0.22)	0.18 (0.15 ; 0.21)	7:35 (6:51 ; 8:17)
	♂ *Lxrα*^*-/-*^	0.23 (0.19 ; 2.66)	0.18 (0.14 ; 0.24)	7:48 (6:44 ; 8:54)
	♀ WT	0.43 (0.35 ; 0.52)*	0.56 (0.44 ; 0.69)*	5:45 (4:53 ; 6:35)*
	♀ *Lxrα*^*-/-*^	0.29 (0.22 ; 0.36)	0.38 (0.28 ; 0.48)*	4:55 (3:56 ; 5:54)*

For each parameter measured, values are represented as median ± 95% bootstrap confidence intervals.

* indicates significant differences between females *vs* males

† indicates significant differences between *Lxrα*^*-/-*^ *vs* WT mice.

Circadian rhythmicity was considered significant for a *p*-value < 0.05; NSR, not significantly rhythmic.

**Fig 5:**
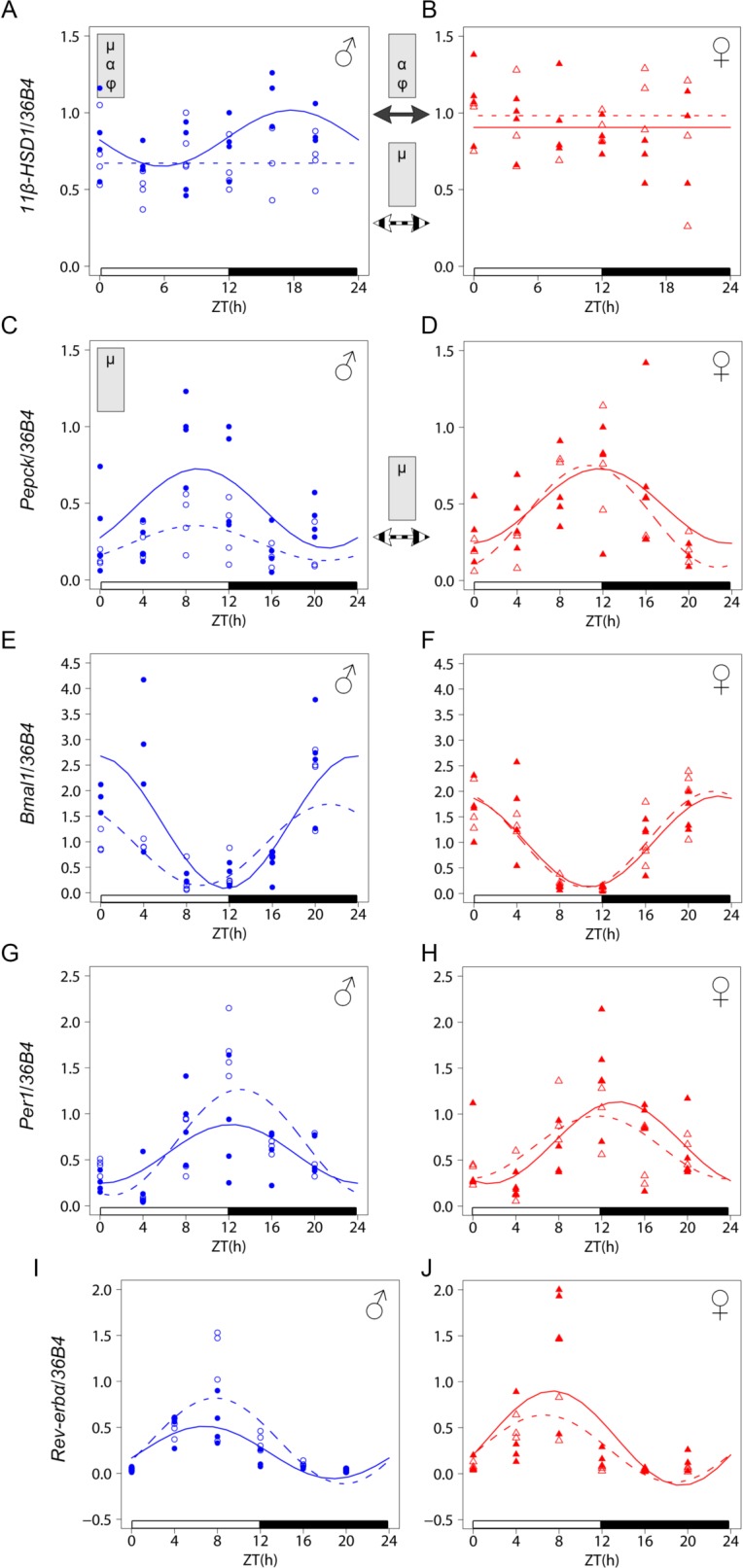
Analysis of circadian gene expression in the liver of WT and *Lxrα*^*-/-*^ *mice*. Diurnal mRNA expression of liver *11β-HSD1* (A, B), *Pepck* (C, D), *Bmal1* (E, F), *Per1* (G, H) and *Rev-erbα* (I, J) was compared using qRT-PCR in WT (plain line) and *Lxrα*^*-/-*^ mice (dashed line). For each time point, 3–4 mice were used. Cosinor-based non-linear regression was used for curve fitting. The ZT0 time point is double plotted for visualization purpose. Expression data were normalized to the constitutively expressed *36B4* mRNA. The white and black bars represent the light and dark phases, respectively. Statistically significant differences in cosine fitting parameters (p<0.05) between wild type and *Lxrα*^*-/-*^ mice or between male and female of the same genotype is indicated in the grey box at the top of the corresponding graph or between graphs (WT: plain arrow, *Lxrα*^*-/-*^: dashed arrow). μ, α and φ indicate a difference in mean level, amplitude and acrophase, respectively.

To extend our analysis of possible LXR dependent sexual dimorphism in circadian regulation, we also analyzed clock gene expression in both the adrenals and the liver where LXR*α* has a prominent role. Our analysis focused on *Per1*, *Bmal1* and *Rev-erbα*, three key components of the molecular clock. Differences were observed in the adrenals between WT males and females for both *Per1* and *Rev-erbα* mean level and acrophase, while *Bmal1* pattern did not differ between sexes (Fig 4E–4J and Table 4). Strikingly, we found that LXR*α* deficiency resulted in an arrhythmic *Per1* expression in males while females showed no significant changes. By contrast, a trend for a decreased *Rev-erbα* mean level was only seen in *Lxrα*^*-/-*^ females, so that they were not different from *Lxrα*^*-/-*^ males any more. The *Per1*, *Bmal1* and *Rev-erbα* expression profiles were similar in the liver irrespective of sex or genotype (Fig 5E–5J, Table 5).

**Table 5:** Cosinor analysis of circadian gene expression in the liver of WT and *Lxrα*^*-/-*^ mice.

Gene	Group	Mean level	Amplitude	Acrophase (ZT:min)
*11β-HSD1*	♂ WT	0.83 (0.76 ; 0.91)	0.18 (0.09 ; 0.29)	17:27 (15:23 ; 20:01) †
	♂ *Lxrα*^*-/-*^	0.67 (0.60 ; 0.74)†	NSR	NSR
	♀ WT	0.90 (0.83 ; 0.98)	NSR	NSR
	♀ *Lxrα*^*-/-*^	0.98 (0.84 ; 1.14) *	NSR	NSR
*Pepck*	♂ WT	0.47 (0.36 ; 0.58)	0.26 (0.12 ; 0.43)	9:06 (6:21 ; 11:21)
	♂ *Lxrα*^*-/-*^	0.24 (0.19 ; 0.30)†	0.12 (0.04 ; 0.19)	8:33 (6:12 ; 11:24)
	♀ WT	0.49 (0.38 ; 0.61)	0.24 (0.10 ; 0.41)	11:24 (8:33 ; 14:19)
	♀ *Lxrα*^*-/-*^	0.42 (0.34 ; 0.50)*	0.34 (0.23 ; 0.45)	10:25 (9:14 ; 12:01)
*Per1*	♂ WT	0.56 (0.43 ; 0.71)	0.32 (0.15 ; 0.52)	12:12 (9:38 ; 14:30)
	♂ *Lxrα*^*-/-*^	0.69 (0.54 ; 0.85)	0.58 (0.39 ; 0.79)	13:01 (11:37 ; 14:29)
	♀ WT	0.69 (0.53 ; 0.85)	0.45 (0.24 ; 0.68)	13:19 (11:15 ; 15:19)
	♀ *Lxrα*^*-/-*^	0.64 (0.51 ; 0.76)	0.34 (0.19 ; 0.53)	11:22 (9:21 ; 13:21)
*Bmal1*	♂ WT	1.38 (1.05 ; 1.72)	1.30 (0.86 ; 1.80)	23:41 (22:20 ; 01:00)
	♂ *Lxrα*^*-/-*^	0.94 (0.75 ; 1.15)	0.80 (0.54 ; 1.10)	21:17 (19:55 ; 22:42)
	♀ WT	1.02 (0.84 ; 1.21)	0.89 (0.64 ; 1.17)	22:47 (21:39 ; 23:56)
	♀ *Lxrα*^*-/-*^	1.06 (0.90 ; 1.23)	0.95 (0.72 ; 1.21)	22:20 (21:20 ; 23:17)
*Rev-erbα*	♂ WT	0.23 (0.17 ; 0.29)	0.29 (0.21 ; 0.37)	6:29 (5:25 ; 7:33)
	♂ *Lxrα*^*-/-*^	0.20 (0.10 ; 0.32)	0.31 (0.17 ; 0.47)	7:42 (5:45 ; 9:42)
	♀ WT	0.41 (0.24 ; 0.60)	0.50 (0.26 ; 0 ;78)	7:00 (4:48; 9:03)
	♀ *Lxrα*^*-/-*^	0.27 (0.17; 0.39)	0.37 (0.22; 0.54)	6:37 (4.50; 8:23)

For each parameter measured, values are represented as median ± 95% bootstrap confidence intervals.

* indicates significant differences between females *vs* males

† indicates significant differences between *Lxrα*^*-/-*^ *vs* WT mice.

Circadian rhythmicity was considered significant for a *p*-value < 0.05; NSR, not significantly rhythmic.

Because both the *StAR* and *MC2R* patterns were unlikely to explain the observed CORT pattern in LXR*α* deficient mice, while clock function was not globally altered in the periphery, we additionally explored the expression of hepatic *type 1 11β-hydroxysteroid dehydrogenase* (*11β-HSD1*) which catalyzes the reactivation of 11-dehydrocorticosterone in the periphery and thereby locally controls glucocorticoid signaling [36]. We found that *11β-HSD1* is rhythmically expressed with low amplitude in the liver from WT males but not in females (Fig 5A and 5B and Table 5). The *11β-HSD1* mRNA oscillation was abolished in *Lxrα* ^*-/-*^ males while remaining unchanged in females as compared to WT animals (Fig 5A and 5B and Table 5). We conclude from this gene expression profiling that loss of LXR*α* has a highly selective effect that is restricted to adrenal *Per1* and hepatic *11β-HSD1*in males and *StAR* in females. This data may point to a primary defect both in liver and adrenal in males whereas only adrenal steroidogenesis seems to be affected in females.

### Circadian rhythms of metabolic parameters are differentially impacted in LXR *α* ^-/-^ male and female mice

**Fig 6:**
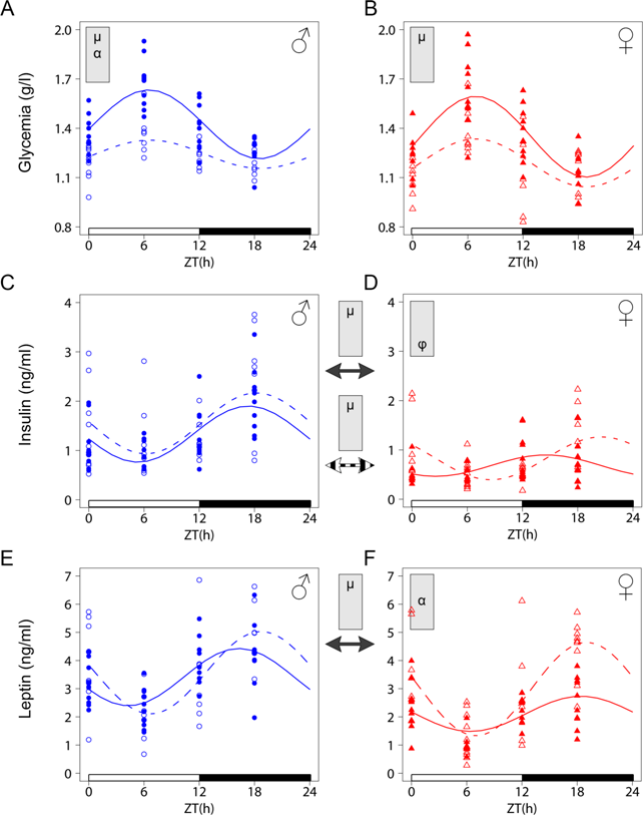
Analysis of circadian variation of glucose, insulin and leptin in WT and *Lxrα*^*-/-*^ mice. Plasma glucose (A, B), insulin (C, D) and leptin (E, F) were determined in WT and *Lxrα*^*-/-*^ mice entrained to a LD12:12 cycle. Cosinor-based non-linear regression was used for curve fitting. The ZT0 time point is double plotted for visualization purposes. The white and black bars represent the light and dark phases, respectively. Statistically significant differences in cosine fitting parameters (p<0.05) between wild type and *Lxrα*^*-/-*^ mice or between male and female of the same genotype is indicated in the grey box at the top of the corresponding graph or between graphs (WT: plain arrow, *Lxrα*^*-/-*^: dashed arrow). μ, α and φ indicate a difference in mean level, amplitude and acrophase, respectively.

Glucocorticoid hormones play a crucial role in metabolic homeostasis and LXR*α* has been established as an important metabolic sensor in mammals. We therefore sought to link the observed selective changes in CORT levels and circadian gene expression to physiological parameters related to glucose homeostasis. We first analyzed plasma glucose levels at 4 time points along the 24 h cycle and observed an approximately two-fold decrease of amplitude in *Lxrα*^*-/-*^ males as compared to their controls, leading to hypoglycemia during all the resting phase (Fig 6A and 6B, Table 6). *Lxrα*^*-/-*^ females were also affected but to a lesser extent. Hepatic glycogen content measured at ZT0 and ZT12 varied as expected but revealed no effect of sex or genotype (S3A and S3B Fig). However, we noticed that the sex difference in food intake was abolished in *Lxrα* ^*-/-*^ mice (S3C Fig).

**Table 6:** Cosinor analysis of circadian variation of glucose, insulin and leptin in WT and *Lxrα*^*-/-*^ mice.

Parameter	Group	Mean level	Amplitude	Acrophase (ZT:min)
Glucose	♂ WT	1.42 (1.38 ; 1.46)	0.21 (0.16 ; 0.27)	6:53 (5:27 ; 7:36)
	♂ *Lxrα*^*-/-*^	1.24 (1.21 ; 1.27)†	0.09 (0.04 ; 0.14)†	6:40 (4:23 ; 8:49)
	♀ WT	1.35 (1.30 ; 1.40)	0.25 (0.18 ; 0.32)	6:53 (5:46 ; 7:59)
	♀ *Lxrα*^*-/-*^	1.19 (1.13 ; 1.24)†	0.15 (0.08 ; 0.22)	6:51 (4:34 ; 8:54)
Insulin	♂ WT	1.33 (1.18 ; 1.49)	0.57 (0.36 ; 0.81)	17:20 (15:50 ; 18:49)
	♂ *Lxrα*^*-/-*^	1.55 (1.27 ; 1.85)	0.62 (0.27 ; 1.07)	18:12 (15:26 ; 20:54)
	♀ WT	0.68 (0.57 ; 0.80)*	0.22 (0.08 ; 0.39)	14:34 (10:43 ; 17:32)
	♀ *Lxrα*^*-/-*^	0.83 (0.68 ; 0.98)*	0.44 (0.25 ; 0.67)	20:34 (18:16 ; 22:27)†
Leptin	♂ WT	3.41 (3.14 ; 3.70)	1.01 (0.67 ; 1.40)	16:19 (14:54 ; 17:44)
	♂ *Lxrα*^*-/-*^	3.57 (3.14 ; 4.03)	1.48 (0.86 ; 2.14)	18:43 (17:09 ; 20:23)
	♀ WT	2.10 (1.86 ; 2.36)*	0.62 (0.30 ; 1.03)	18:25 (16:15 ; 20:35)
	♀ *Lxrα*^*-/-*^	2.99 (2.61 ; 3.39)	1.67 (1.19 ; 2.21)†	19:01 (17:34 ; 20:20)

For each parameter measured, values are represented as median ± 95% bootstrap confidence intervals.

* indicates significant differences between females *vs* males

† indicates significant differences between *Lxrα*^*-/-*^ *vs* WT mice.

Circadian rhythmicity was considered significant for a *p*-value < 0.05; NSR, not significantly rhythmic.

To determine whether changes in glycemia could be caused by a differential hepatic glucose production, we monitored the expression of the *phosphoenolpyruvate carboxykinase* (*Pepck*) gene, which encodes a rate limiting step in the hepatic gluconeogenesis pathway. Results showed a significant decrease in the amplitude of *Pepck* mRNA oscillation in *Lxrα* ^*-/-*^ males as compared to controls while females from both genotypes exhibited a similar circadian expression profile. (Fig 5C and 5D and Table 5). At the physiological level, plasma insulin, oscillated in both males and females but with higher amplitude in males. This profile was not altered in *Lxrα* ^*-/-*^males while females displayed a 6 hours phase delay as compared to WT animals (Fig 6C and 6D and Table 6). Because leptin is known to regulate glucose metabolism through its action in the brain, we also investigated its circadian pattern in the *Lxrα* ^*-/-*^ model. We found that WT females displayed lower mean levels than WT males. In contrast, this sexual dimorphism was not observed in *Lxrα* ^*-/-*^ animals because *Lxrα* ^*-/-*^ females exhibited significantly higher amplitude of the leptin rhythm thus resembling males (Fig 6E and 6F and Table 6). These data collectively indicate that the circadian regulation of metabolic parameters is altered in a sex-specific manner in LXR*α* deficient mice.

## Discussion

LXR*α* is an essential metabolic sensor which upon binding to oxysterols regulates the transcription of genes critical for cholesterol, lipid, and glucose homeostasis [24]. In this report we identified a novel and unanticipated role for LXR*α* as a determinant of the sexual dimorphism of circadian physiology. *Lxrα*^*-/-*^ male mice have previously been reported to display adrenomegaly and increased plasma CORT levels, although the circadian pattern was not investigated [26]. We and others reported significant sex specific differences in the circadian rhythm of plasma CORT which show higher levels and amplitude in females than in males [29, 37, 38]. The observation that CORT was arrhythmic in *Lxrα*^*-/-*^ males while females displayed higher amplitude is of importance because there is compelling evidence from the literature that glucocorticoid hormones are potent synchronizers of peripheral circadian clocks in addition to their multiple physiological roles [16, 39–41]. For instance, phase-shifting the CORT pattern using metyrapone changed the speed of behavioral re-entrainment after a jetlag and transplanting clock-deficient adrenals in adrenalectomized WT hosts caused a faster re-entrainment after a 6-hour phase advance [33]. Furthermore it was shown in rat that CORT rhythmic secretion is critical for normal resetting behavior [34]. We hypothesize that the faster re-entrainment seen in *Lxrα*^*-/-*^ males may result at least in part from the altered CORT pattern. The circadian rhythm of CORT secretion by the adrenal cortex is regulated both at the central level by the SCN and locally through the HPA axis. Because ACTH levels are unchanged in *Lxrα*^-/-^ mice while adrenals from these animals secrete more CORT *in vitro*, Cummins *et al* excluded a dysregulation of the HPA axis in these animals [26]. At the adrenal level, the rhythmic secretion pattern of GCs is determined by both the sensitivity to ACTH and GCs biosynthesis rate. Accordingly, the adrenal responsiveness to ACTH follows a diurnal rhythm, with a higher sensitivity during the activity phase in rodents [42, 43]. The *StAR* gene that encodes a rate-limiting enzyme of the steroidogenic pathway is the only component showing a robust circadian expression in the adrenal [44]. Expression of the *MC2R* and *StAR* mRNA was circadian and sexually dimorphic in our study, but this was not changed in *Lxrα* ^*-/-*^ mice suggesting that the damped CORT pattern observed in *Lxrα* ^*-/-*^ males may primarily result from the adrenomegaly reported by Cummins et al [26].

Notably, the biological effects of GCs involve to a large extent the *11β-HSD1* enzyme that catalyzes the reduction of plasma 11-dehydrocorticosterone to CORT. This reactivation pathway plays a major role for local production of CORT and can also indirectly impact adrenal CORT secretion [36, 45, 46]. The liver, which is the main site of LXR*α* expression, also contains the highest concentration of *11β-HSD1*. Mice lacking *11β-HSD1* display compensatory adrenomegaly and elevated morning (diurnal nadir) plasma CORT concentrations but similar peak levels [45]. Intriguingly, this phenotype is highly reminiscent to that observed in *Lxrα*^*-/-*^ male mice and prompted us to investigate hepatic *11β-HSD1*expression around the clock. We found that the circadian pattern of *11β-HSD1* mRNA expression was totally damped in *Lxrα*^*-/-*^ males. An earlier study failed to detect this change possibly because only one time point in the early light phase was analyzed [47]. Yet this previous paper reported a decreased hepatic expression of *11β-HSD1* upon treatment with the LXR agonist TO901317 [47]. The similar effect of both the agonist treatment and the lack of LXR*α* on *11β-HSD1* expression can be explained by the fact that the apoLXR*α* is a repressor which, upon deletion or activation, induces an intermediary repressor of the *11β -HSD1* gene [47]. Altogether, this data strongly suggest that LXR*α* regulates the circadian rhythm of GCs production in males both at the systemic and local levels through its action on the adrenals and *11β -HSD1* respectively. Presumably, females which exhibit higher CORT levels and amplitude than males do not require further circadian time dependent increase of the local regeneration of CORT. This may explain why *11β -HSD1* is neither rhythmic nor regulated by LXR*α* in females. Interestingly, mice lacking *11β -HSD1* are hypoglycemic and a have decreased *Pepck* response to fasting hypoglycemia [45]. Thus the lower amplitude of plasma glucose oscillation seen in *Lxα*^*-/-*^males may result, at least in part, from an altered stimulation of the *Pepck* gene by locally produced GCs. This demonstrates that LXR*α* is required not only for normoglycemia but also for normal daily oscillation of glucose levels. We do not exclude that this role in the interaction between glucose homeostasis and circadian timing could also involve LXR*α* outside the liver. Indeed insulin sensitivity is known to be under control of the SCN clock and consequently displays daily variations [48]. Activation of LXRs using the GW3965 agonist improves glucose tolerance in a mouse model of diet-induced obesity and insulin resistance [49]. These effects are attributable to the role of LXR*α* in insulin secretion and glucose uptake. It is therefore plausible that the damped plasma glucose profile in *Lxrα*^*-/-*^ males also originates from a time specific increase (ZT0-ZT12) of peripheral sensitivity to insulin resulting from the loss of LXR*α* [49, 50]. Although female LXR*α* mice did not show significant changes in their plasma glucose profile, there was also a trend toward decreased levels. Additionaly, they displayed a dramatic phase delay of their insulin profile (6 hours) that can be interpreted as a consequence of the significant increase in the amplitude of leptin, an adipokine showing a circadian variation in plasma and regulated by GCs[51, 52]. The dramatic increase in the amplitude of CORT in females could therefore explain the gender specific increase in the amplitude of leptin rhythmicity, resulting in a higher demand for insulin secretion.

LXR*α* not only influences the production of corticosteroids but also that of sex steroids. *Lxrα*^*-/-*^ female mice display elevated levels of 17β-estradiol and an ovarian hyperstimulation phenotype [53]. Unexpectedly, expression of *Cyp19* which aromatizes androgens was found to be decreased while that of *Cyp11a1* was increased in *Lxrα*^*-/-*^ female mice, suggesting that they may also produce more testosterone than WT animals. Males lacking LXR*α* show a contrasting situation as they display decreased levels of testicular testosterone associated to an increased apoptosis rate of germ cells [54]. These effects of LXR*α* on sex steroid production could therefore also contribute the sexual dimorphism of physiological parameters including those under circadian variation. Importantly, there is evidence that the gonadotropic axis and glucocorticoid signaling interfere at different levels. GCs are known to directly inhibit gonadotropin-stimulated testosterone production [55], and serum corticosterone and testosterone levels are inversely correlated [56]. Consistently, chronic corticotherapy reduces serum testosterone levels In human patients [57]. Crosstalk between the estrogen, GCs and LXR pathways have been described. [58]. [59, 60]. Collectively, this and our data suggests that loss of LXR*α* compromises at the organismal level the fine tuning of the sex-specific balance between glucocorticoid, estrogen and androgen production and action.

The LXR*α* dependent co-regulation of clock outputs is another mechanism that could also underlie the observed phenotype. For instance LXR*α* shares many targets with the circadian nuclear receptor PPAR*α* and the formation of LXR*α*-PPAR*α* heterodimer has been shown to downregulate *Cyp7α1* expression [61, 62]. LXR*α* is also positively regulated by the deacetylase SIRT1, an enzyme displaying a circadian activity in liver [63, 64]. Thus, LXR*α* activity could be modulated posttranslationally by the circadian clock through rhythmic deacetylation.

We excluded a direct role of LXR*α* in the transcriptional regulation of the clock network because *Per1* was the only core clock gene found to be changed in *Lxrα*^*-/-*^ animals and this was restricted male adrenals. This suggests that *Per1* rhythmic expression in the adrenal is more likely to be driven by a systemic cue such as CORT, the production of which is altered in *Lxrα*^*-/-*^ males (see below). This hypothesis is supported by the observation that *Per1* is an hyper-responsive gene to GCs [65]. In addition two previous genome wide studies failed to identify core clock components among genes regulated by the LXR*α* agonist T0901317 or directly bound by LXR*α* [61, 66]. Finally the marginal effects of the LXR*α* mutation on the free-running period of locomotor activity also supports that LXR*α* does not play a significant role in core clock mechanism.

Despite a considerable male bias in most animal studies [67], sexual dimorphism in metabolic and circadian physiology has been recognized in mammals [68, 69]. Reproductive factors including sex hormones are considered as major determinants of such differences but non-reproductive factors including glucocorticoid hormones also appear to play a significant role [70]. Compelling evidences have recently linked metabolic homeostasis and circadian timing and involved numerous nuclear hormone receptors. We extend this concept by linking the metabolic nuclear receptor LXR*α* to the circadian and sex-dependent regulation of physiology. These findings are of importance in the context of pharmacological studies or future personalized therapies targeting LXR*α*.

## Materials and Methods

### Animals

Mutant *Lxrα*^*-/-*^ breeders in the C57BL/6j background were obtained from Taconic and subsequently crossed in our facility. Control C57BL/6j animals were from Charles River (France) and adapted to the facility environment for at least 6 weeks before the experiments to prevent any bias related to housing conditions. Mice were housed in a temperature-controlled room with a 12-hours light (325 lux)/12-hours dark (LD 12:12) cycle and fed *ad libitum*. All experiments were performed with 3 months-old animals. Animal experiment procedures were carried out in accordance with the CNRS and INSERM institutional guidelines. The local ethical committee (Comité Institutionnel d'Éthique Pour l'Animal de Laboratoire CIEPAL-AZUR PEA N° NCE 2011–26) specifically approved this study.

### Plasma metabolic parameters measurements

Blood samples were collected from the retro-orbital venous plexus in heparin containing tubes, and plasma was separated by centrifugation for 20 min at 3,000 rpm. For each mouse, blood was collected only twice (two different ZT times), at 2 weeks intervals, to avoid stress effects. Plasma glucose was determined using an Accu-Check glucometer (Roche Diagnostics, France). Serum insulin, leptin and corticosterone concentrations were measured with commercial enzyme-linked immunosorbent assays from respectively Mercodia (Uppsala, Sweden) R&D (MOB00) and Molecular Devices companies with an enzyme standard instrument for serum corticosterone levels, according to the manufacturer’s recommendations.

### RNA extraction and quantitative real-time (qRT)-PCR

Total RNA was extracted using the single step method described by Chomczynski andmRNA levels were measured by real-time (RT)–qPCR using a Light Cycler 1.5 (RocheApplied Science) and SYBR green I dye detection according to the manufacturer's recommendation. cDNA, synthesized from 2 to 5 μg of total RNA using random primers and Superscript II (Invitrogen), was added to a reaction mixture (Faststart DNA SYBR green I; Roche Diagnostics) with appropriate primers at 0.5 mM each (*Lxrα*: forward 5’-CTGATGTTTCTCCTGATTCTGC-3’ and reverse 5’-CTTTTTCCGCTTTTGTGGAC-3’, *Cyp7α1*: forward 5’-TACTTCTGCGAAGGCATTTGG-3’ and reverse 5’-TACTTCTGCGAAGGCATTTGG-3’, *StAR*: forward 5’-AGGAAAGCCAGCAGGAGAAC-3’ and reverse 5’-TGATGACCGTGTCTTTTCCA-3’, *11β-HSD1*: forward 5’-GGCGGGAAAGCTCATGG-3’ and reverse 5’-AAGGAGGAGATGACGGCAAT-3’, *MC2R*: forward 5’-GCCCTTCTAAGCCAGATC-C-3’ and reverse 5’-ATTTCTTGCGGTGTCATTGG-3’, *Per1*: forward 5’-GAAGTTTGAGCTCCCGAAGT-3’ and reverse 5’-TGAGAGCAGCAAGAGTACAAAC-3’, *Rev-erbα*: forward 5’-AACCTCCAGTTTGTGTCAAGGT-3’ and reverse 5’-GATGACGATGATGCAGAAGAAG-3’ *Bmal1*: forward 5’-CTCATTGATGCCAAGACTGG-3’ and reverse 5’-GGTGGCCAGCTTTTCAAATA-3’, *Pepck*: forward 5’-TTTGATGCCCAAGGCAACTT-3’ and reverse 5’-ATCGATGCCTTCCCAGTAAA-3’, *36B4*: forward 5’-GCTGATGGGCAAGAACACCA-3’ and reverse 5’-CCCAAAGCCTGGAAGAAGGA-3’). The relative mRNA abundance was calculated using a standard-curve method. Expression levels were normalized to the levels of the constitutively expressed 36B4 ribosomal protein mRNA.

### Running wheel activity measurement

Mice were individually housed within light-controlled isolation chambers in cages containing monitored activity wheels, and they were allowed *ad libitum* access to food and water. Animals were weighed weekly and their daily food intake in each condition was estimated by measuring the difference between the quantity of food provided and food remaining after 1 week / 7. Mice were entrained to an initial 12:12 LD cycle (Light phase from 7:00 to 19:00, ZT0 = lights on = 7:00). Daily as well as (subjective) day and night activity were quantified using Clocklab plugin for Matlab (Actimetrics). After 3 weeks of activity recording on this cycle, they were transferred to constant darkness (DD). Their free running period was assessed based on activity onset on the second week of DD using Clocklab. Mice were then returned to the initial 12:12 LD cycle for re-entrainment. After 3 weeks, animals were subjected to an 8h phase shift (Jetlag experiment) by advancing the light phase from 23:00 to 11:00. The time to re-entrain to the new lighting schedule was determined based on activity onset and acrophase using Clocklab.

### Statistical analysis

Activity data are reported as median ± 95% bootstrap confidence intervals. For the jetlag experiment, results are represented as boxplots. Data were analysed using a Kruskal and Wallis rank sum test for multiple comparison between groups followed by non-parametric pairwise *post-hoc* test. For time series, data was modelled using a non-linear regression (Cosine fitting analysis: a + b * cos(2 * pi * (ZT—c)/24); where a = mean expression level, b = amplitude of the oscillation and c = acrophase). Cosine fitting analysis was followed by bootstrapping to compare confidence intervals on a, b and c parameters between time series. Significance level was set at p < 0.05. Statistical analysis was performed using the R software (version 2.15.2; The R Foundation for Statistical Computing).

## Supporting Information

S1 Fig Analysis of *GR* expression in the liver. Diurnal mRNA expression of liver *GR* was compared in males (blue) and females (red) using qRT-PCR in WT and *Lxrα*^*-/-*^ mice. For each time point, 3–4 mice were used. Cosine-based non-linear regression was used for curve fitting. The ZT0 time point is double plotted for visualization purposes. Expression data were normalized to the constitutively expressed *36B4* mRNA. The white and black bars represent the light and dark phases, respectively.Statistically significant differences in cosine fitting parameters (p<0.05) between wild type and *Lxrα*^*-/-*^ mice or between male and female of the same genotype is indicated in the grey box at the top of the corresponding graph or between graphs (WT: plain arrow, *Lxrα*^*-/-*^: dashed arrow). μ, α and φ indicate a difference in mean level, amplitude and acrophase, respectively.
(TIF)

S2 Fig Analysis of *Lxrα* expression in the adrenal. Diurnal mRNA expression of adrenal *Lxrα* was compared in males (blue) and females (red) using qRT-PCR in WT mice. For each time point, 3–4 mice were used. Cosine-based non-linear regression was used for curve fitting. The ZT0 time point is double plotted for visualization purposes. Expression data were normalized to the constitutively expressed *36B4* mRNA. The white and black bars represent the light and dark phases, respectively. Statistically significant differences in cosine fitting parameters (p<0.05) between wild type and *Lxrα*^*-/-*^ mice or between male and female of the same genotype is indicated in the grey box at the top of the corresponding graph or between graphs (WT: plain arrow, *Lxrα*^*-/-*^: dashed arrow). μ, α and φ indicate a difference in mean level, amplitude and acrophase, respectively.
(TIF)

S3 Fig Food intake and liver glycogen content for each gender and each genotype. (A) Mean daily food intake (g) in male and female WT and *Lxrα*^*-/-*^ mice. Note there is a significant difference between WT males and WT females (p>0.05). (B-C) Glucose content liberated from glycogen in liver pieces at ZT0 and ZT12. (B) Female WT vs *Lxrα*^*-/-*^ mice (n = 4) and (C) male WT vs *Lxrα*^*-/-*^ mice (n = 4). There is a significant difference in liver glycogen content between ZT0 and ZT12 (p>0.0001) but no influence of the LXRα mutation.
(TIF)

S1 File Glycogen assay. Liver pieces collected at ZT0 and ZT12, from 4 animals of each group were used in this experiment. For each sample, 30 mg of liver was lysed in KOH 0.5M at 95°C. Na_2_SO_4_ 6% (25 μl) and 750 μl methanol were then added. Glycogen was precipitated at -80°C in 2 separate aliquots for each sample. After centrifugation, glycogen was either resuspended in 200 μl amyloglucosidase 2 mg/ml (Sigma-Aldrich) or in 200 μl sodium acetate, to assay total glucose and free glucose respectively. Suspensions were incubated for 1-h at 37°C. Free/total glucose content was measured on 5 μL of supernatant in 300 μl of reagent using a glucose hexokinase assay kit (Sigma-Aldrich) according to manufacturer’s protocol. Glucose was expressed in μmol/g wet liver. Glucose coming from glycogen was determined as (total glucose)-(free glucose) in each sample.
(DOCX)

S1 Table Cosinor analysis of *GR* circadian expression in WT and *Lxrα-/-* mice. Values are represented as median ± 95% bootstrap confidence intervals. * indicates significant differences between females vs males, † indicates significant differences between *Lxrα-/- vs* WT mice. Circadian rhythmicity was considered significant for a *p*-value < 0.05; NSR, not significantly rhythmic.
(DOCX)
