# Supplementary figures and images for: Sexual Dimorphism in Circadian Physiology Is Altered in LXRα Deficient Mice
Source: PLoS One. 2016 Mar 3;11(3):e0150665. doi: 10.1371/journal.pone.0150665 (PMC4777295; doi:10.1371/journal.pone.0150665)

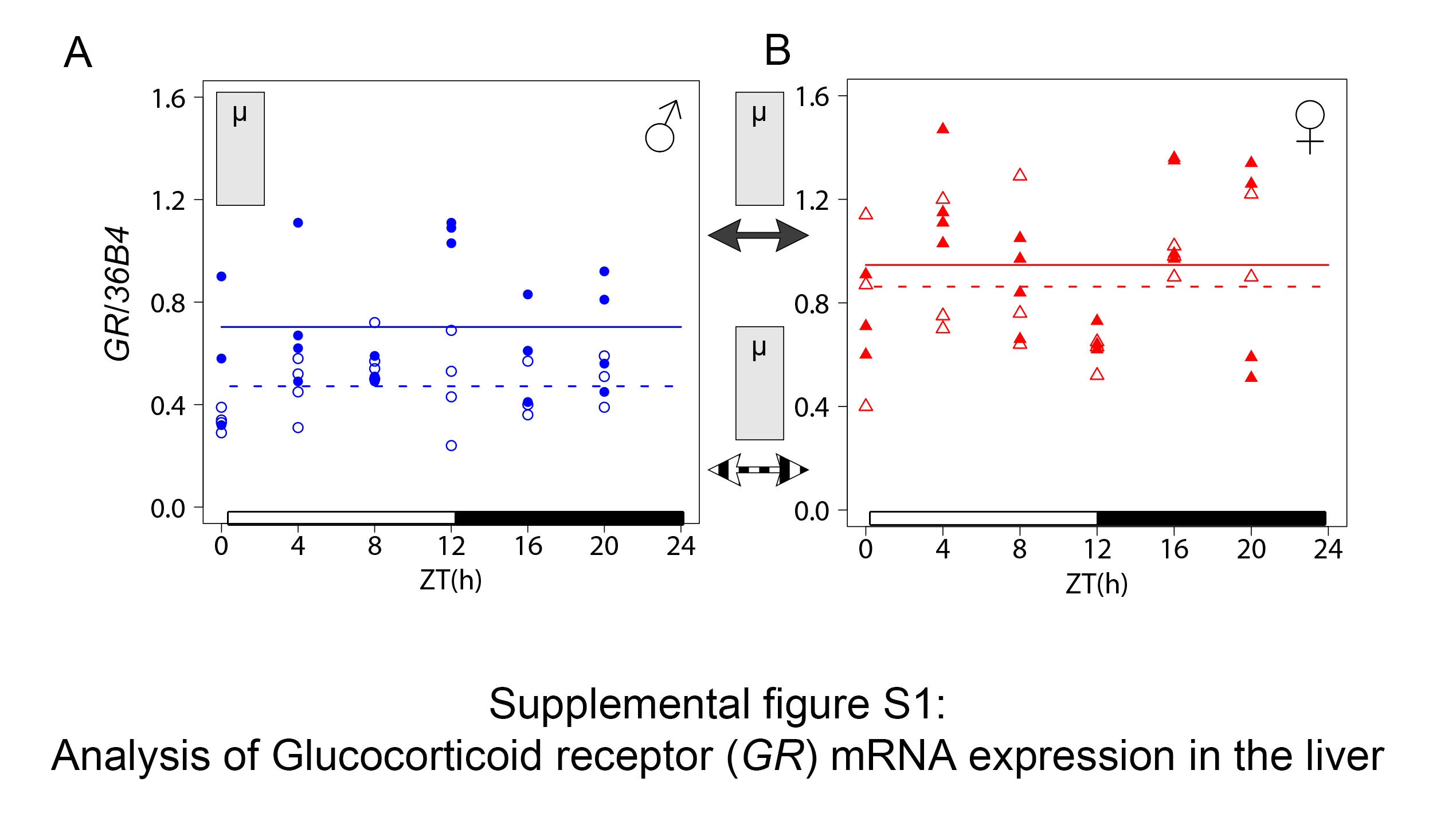

Supplement: S1 Fig — Diurnal mRNA expression of liver GR was compared in males (blue) and females (red) using qRT-PCR in WT and Lxrα-/- mice. For each time point, 3–4 mice were used. Cosine-based non-linear regression was used for curve fitting. The ZT0 time point is double plotted for visualization purposes. Expression data were normalized to the constitutively expressed 36B4 mRNA. The white and black bars represent the light and dark phases, respectively.Statistically significant differences in cosine fitting parameters (p<0.05) between wild type and Lxrα-/- mice or between male and female of the same genotype is indicated in the grey box at the top of the corresponding graph or between graphs (WT: plain arrow, Lxrα-/-: dashed arrow). μ, α and φ indicate a difference in mean level, amplitude and acrophase, respectively. (TIF) [file pone.0150665.s001.tif]

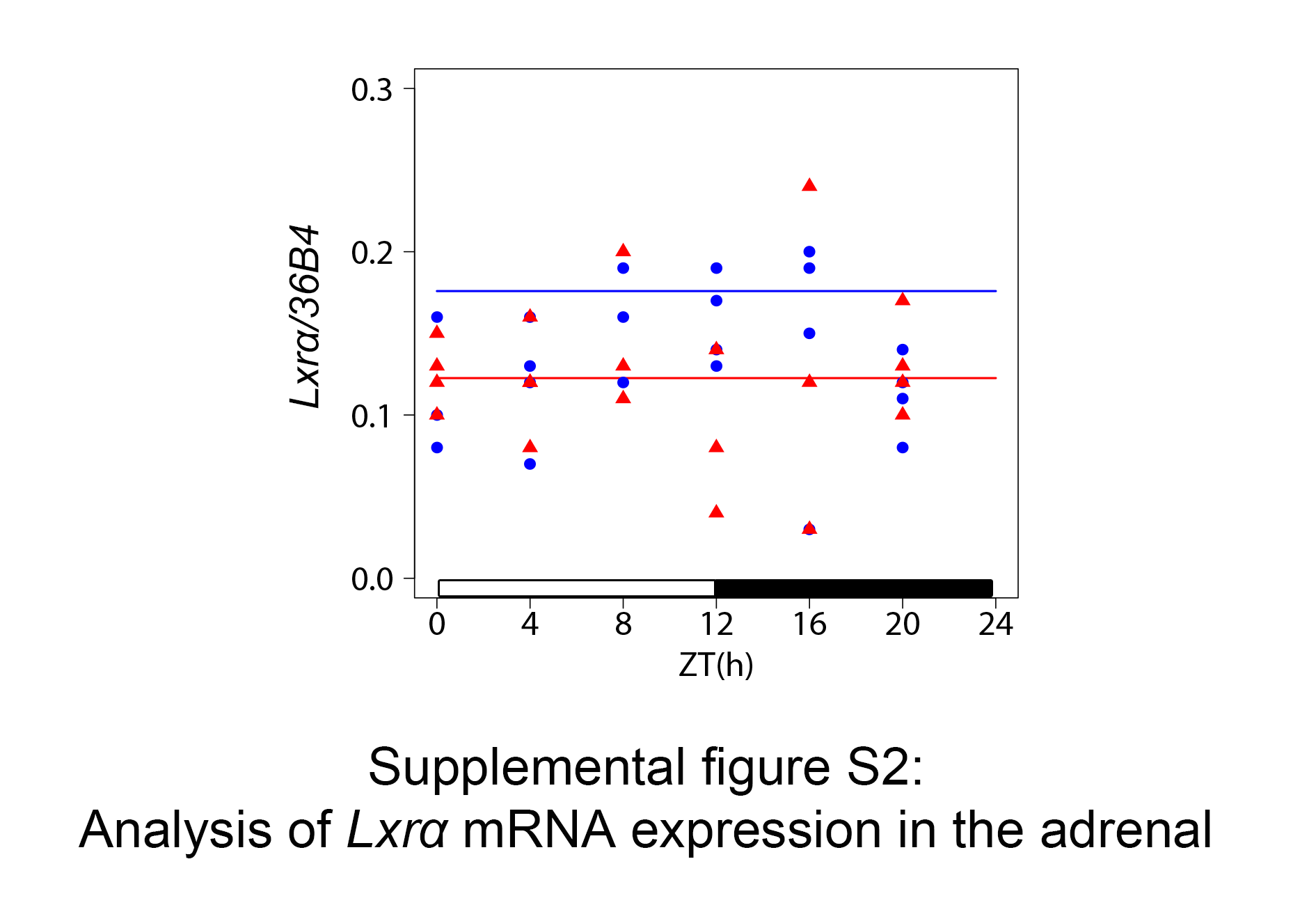

Supplement: S2 Fig — Diurnal mRNA expression of adrenal Lxrα was compared in males (blue) and females (red) using qRT-PCR in WT mice. For each time point, 3–4 mice were used. Cosine-based non-linear regression was used for curve fitting. The ZT0 time point is double plotted for visualization purposes. Expression data were normalized to the constitutively expressed 36B4 mRNA. The white and black bars represent the light and dark phases, respectively. Statistically significant differences in cosine fitting parameters (p<0.05) between wild type and Lxrα-/- mice or between male and female of the same genotype is indicated in the grey box at the top of the corresponding graph or between graphs (WT: plain arrow, Lxrα-/-: dashed arrow). μ, α and φ indicate a difference in mean level, amplitude and acrophase, respectively. (TIF) [file pone.0150665.s002.tif]

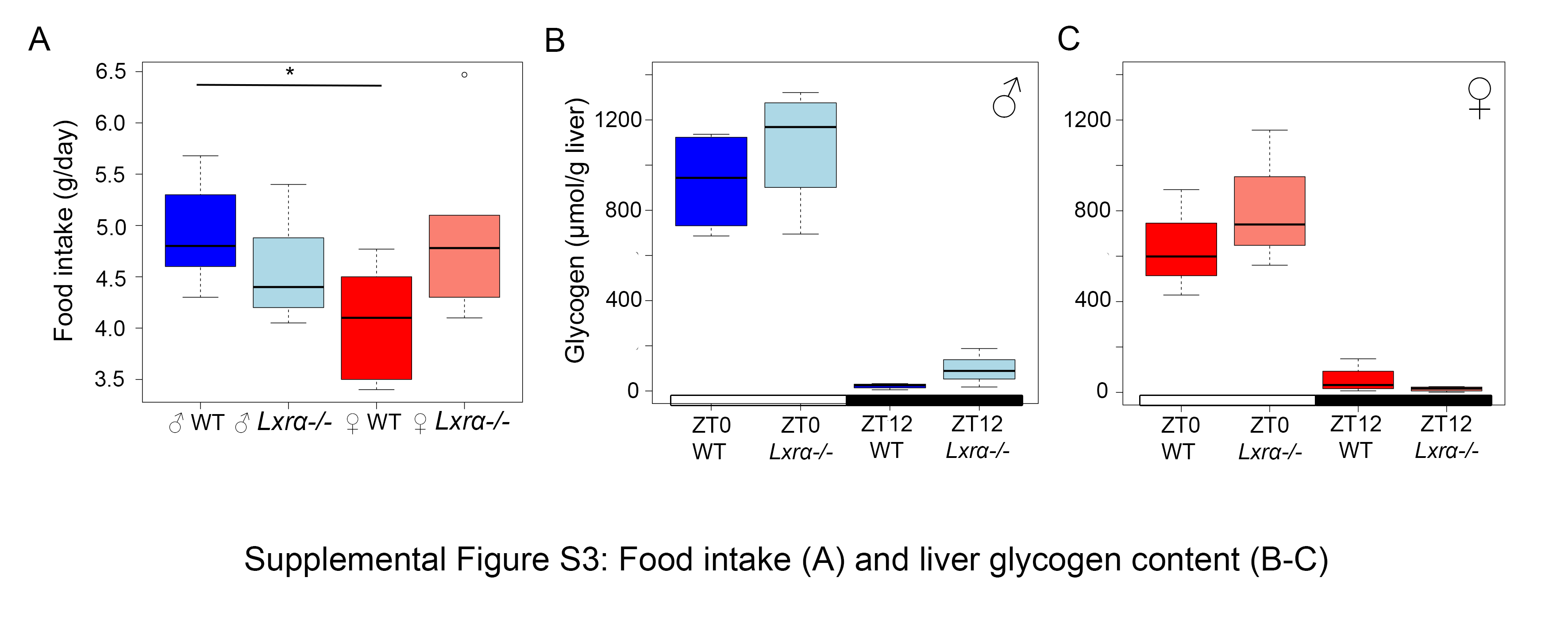

Supplement: S3 Fig — (A) Mean daily food intake (g) in male and female WT and Lxrα-/- mice. Note there is a significant difference between WT males and WT females (p>0.05). (B-C) Glucose content liberated from glycogen in liver pieces at ZT0 and ZT12. (B) Female WT vs Lxrα-/- mice (n = 4) and (C) male WT vs Lxrα-/- mice (n = 4). There is a significant difference in liver glycogen content between ZT0 and ZT12 (p>0.0001) but no influence of the LXRα mutation. (TIF) [file pone.0150665.s003.tif]
