# Supplementary material for: Sexual Dimorphism in Circadian Physiology Is Altered in LXRα Deficient Mice
Source: PLoS One. 2016 Mar 3;11(3):e0150665. doi: 10.1371/journal.pone.0150665 (PMC4777295; doi:10.1371/journal.pone.0150665)
Supplement: S1 File — Liver pieces collected at ZT0 and ZT12, from 4 animals of each group were used in this experiment. For each sample, 30 mg of liver was lysed in KOH 0.5M at 95°C. Na2SO4 6% (25 μl) and 750 μl methanol were then added. Glycogen was precipitated at -80°C in 2 separate aliquots for each sample. After centrifugation, glycogen was either resuspended in 200 μl amyloglucosidase 2 mg/ml (Sigma-Aldrich) or in 200 μl sodium acetate, to assay total glucose and free glucose respectively. Suspensions were incubated for 1-h at 37°C. Free/total glucose content was measured on 5 μL of supernatant in 300 μl of reagent using a glucose hexokinase assay kit (Sigma-Aldrich) according to manufacturer’s protocol. Glucose was expressed in μmol/g wet liver. Glucose coming from glycogen was determined as (total glucose)-(free glucose) in each sample. (DOCX) [file pone.0150665.s004.docx]

**Supplemental methods**

**Glycogen assay**

Liver pieces collected at ZT0 and ZT12, from 4 animals of each group were used in this experiment. For each sample, 30 mg of liver was lysed in KOH 0.5M at 95 °C. Na_2_SO_4_ 6% (25 µl) and 750 µl methanol were then added. Glycogen was precipitated at -80°C in 2 separate aliquots for each sample. After centrifugation, glycogen was either resuspended in 200 µl amyloglucosidase 2 mg/ml (Sigma-Aldrich) or in 200 µl sodium acetate, to assay total glucose and free glucose respectively. Suspensions were incubated for 1-h at 37°C. Free/total glucose content was measured on 5 μL of supernatant in 300 µl of reagent using a glucose hexokinase assay kit (Sigma-Aldrich) according to manufacturer’s protocol. Glucose was expressed in μmol/g wet liver. Glucose coming from glycogen was determined as (total glucose)-(free glucose) in each sample.
