# Supplementary material for: Sexual Dimorphism in Circadian Physiology Is Altered in LXRα Deficient Mice
Source: PLoS One. 2016 Mar 3;11(3):e0150665. doi: 10.1371/journal.pone.0150665 (PMC4777295; doi:10.1371/journal.pone.0150665)
Supplement: S1 Table — Values are represented as median ± 95% bootstrap confidence intervals. * indicates significant differences between females vs males, † indicates significant differences between Lxrα-/- vs WT mice. Circadian rhythmicity was considered significant for a p-value < 0.05; NSR, not significantly rhythmic. (DOCX) [file pone.0150665.s005.docx]

**Supplemental table 1. Cosinor analysis of circadian variation of GR in WT and *Lxrα^-/-^* mice**

| **Gene** | **Group** | **Mean level** | **Amplitude** | **Acrophase (ZT:min)** |
| --- | --- | --- | --- | --- |
| *GR* | ♂ WT | 0.70 (0.61 ; 0.80) | NSR | NSR |
|  | ♂ *Lxrα^-/-^* | 0.47 (0.42 ; 0.51*)*† | NSR | NSR |
|  | ♀ WT | 0.95 (0.83 ; 1.07)* | NSR | NSR |
|  | ♀ *Lxrα^-/-^* | 0.86 (0.75 ; 0.98)* | NSR | NSR |

For each parameter measured, values are represented as median ± 95% bootstrap confidence intervals. * indicates significant differences between females *vs* males, † indicates significant differences between *Lxrα^-/-^* *vs* WT mice. Circadian rhythmicity was considered significant for a *p*-value < 0.05; NSR, not significantly rhythmic.
